# Effects of Growth Hormone (GH) Therapy Withdrawal on Glucose Metabolism in Not Confirmed GH Deficient Adolescents at Final Height

**DOI:** 10.1371/journal.pone.0087157

**Published:** 2014-01-30

**Authors:** Flavia Prodam, Silvia Savastio, Giulia Genoni, Deepak Babu, Mara Giordano, Roberta Ricotti, Gianluca Aimaretti, Gianni Bona, Simonetta Bellone

**Affiliations:** 1 Division of Pediatrics, Department of Health Sciences, University of “Piemonte Orientale Amedeo Avogadro”, Novara, Italy; 2 Endocrinology, Department of Clinical and Experimental Medicine, University of Piemonte Orientale, Novara, Italy; 3 I.C.O.S. (Interdisciplinary Center for Obesity Study), Novara, Italy; 4 Laboratory of Human Genetics, Department of Health Sciences, University of Piemonte Orientale, Novara, Italy; University of Catanzaro Magna Graecia, Italy

## Abstract

**Context, objective:**

Growth hormone deficiency (GHD) is associated with insulin resistance and diabetes, in particular after treatment in children and adults with pre-existing metabolic risk factors. Our aims were. i) to evaluate the effect on glucose metabolism of rhGH treatment and withdrawal in not confirmed GHD adolescents at the achievement of adult height; ii) to investigate the impact of GH receptor gene genomic deletion of exon 3 (d3GHR).

**Design, setting:**

We performed a longitudinal study (1 year) in a tertiary care center.

**Methods:**

23 GHD adolescent were followed in the last year of rhGH treatment (T0), 6 (T6) and 12 (T12) months after rhGH withdrawal with fasting and post-OGTT evaluations. 40 healthy adolescents were used as controls. HOMA-IR, HOMA%β, insulinogenic (INS) and disposition (DI) indexes were calculated. GHR genotypes were determined by multiplex PCR.

**Results:**

In the group as a whole, fasting insulin (p<0.05), HOMA-IR (p<0.05), insulin and glucose levels during OGTT (p<0.01) progressively decreased from T0 to T12 becoming similar to controls. During rhGH, a compensatory insulin secretion with a stable DI was recorded, and, then, HOMAβ and INS decreased at T6 and T12 (p<0.05). By evaluating the GHR genotype, nDel GHD showed a decrease from T0 to T12 in HOMA-IR, HOMAβ, INS (p<0.05) and DI. Del GHD showed a gradual increase in DI (p<0.05) and INS with a stable HOMA-IR and higher HDL-cholesterol (p<0.01).

**Conclusions:**

In not confirmed GHD adolescents the fasting deterioration in glucose homeostasis during rhGH is efficaciously coupled with a compensatory insulin secretion and activity at OGTT. The presence of at least one d3GHR allele is associated with lower glucose levels and higher HOMA-β and DI after rhGH withdrawal. Screening for the d3GHR in the pediatric age may help physicians to follow and phenotype GHD patients also by a metabolic point of view.

## Introduction

Growth hormone (GH) has pleiotropic functions in humans. GH/insulin-like growth factor-1 (IGF-I) axis is the main regulator of post-natal growth, but it has other main metabolic actions such as the regulation of body composition, muscle and bone metabolism. Furthermore, in the post-absorptive state, GH mainly acts on stimulating lipolysis and lipid oxidation in order to switch metabolism from glucose and protein to lipid utilization. At present, it is reported that GH administration is followed by lipolysis but also by insulin resistance and relatively sustained hyperglycemia [1]–[3]. GH-induced lipolysis appears as the most important determinant of GH anti-insulin actions, by inhibiting insulin-stimulated glucose uptake especially in muscles [4], [5]. Whether the impairment in peripheral insulin sensitivity is mainly located in muscle and mostly due to higher disposable free fatty acids, GH is also able to reduce hepatic insulin sensitivity in healthy humans and to counterbalance the anti-lipolytic actions of hyperinsulinemia [1], [6]. Some of these effects are direct actions, whereas others are IGF-I mediated [1].

Other mechanisms may be implicated on the metabolic effects of GH, as the interaction of GH with the insulin receptor [7], [8] and the presence of several polymorphisms including the GH receptor (GHR) exon 3 deletion (d3GHR) which seems to play a role in glucose homeostasis in GHD subjects [9] and in general population [10], [11].

Several studies in adults have determined the effects of GH replacement therapy on insulin sensitivity. Short-term rhGH therapy deteriorates insulin sensitivity with an improvement on long-term in the majority but not in all studies. These conflicting data are probably due to differences in sample size, methods of evaluation of insulin sensitivity and doses of rhGH [1], [12]–[15]. There are suggestions that rhGH therapy increases the risk of type 2 and/or secondary diabetes in GHD adults with pre-existing metabolic risk factors [16]. Similarly, in children data from registries show a slower increase in the incidence of diabetes due to GH treatment in those patients with pre-existing risk factors [17], [18]. Despite this, a few data describe insulin secretion at final height in the “so called” transition phase. Evidence to date suggests an increase in insulin sensitivity after cessation of GH treatment [19]–[21], but more studies are needed to clarify. In particular, no attention has been paid on those subjects which were not reconfirmed as GHD after stopping therapy.

Euglycemic hyper-insulinemic clamp is considered as the “gold standard” for quantifying insulin sensitivity in vivo, but this method is not applicable on a great population in daily clinical practice. Insulinogenic index (INS) and disposition index (DI) are new indirect methods for measure beta-cell function, using oral glucose tolerance test (OGTT) -derived measures, and are early markers of inadequate beta-cell or peripheral compensation. They allow the investigation of a larger number of patients [22]. Recent data indicate INS and DI as the best predictors of future type 2 diabetes in adults [23]–[25].

In order to understand the metabolic effects of rhGH therapy in adolescents with unconfirmed GHD at the achievement of adult height, we evaluated glucose metabolism in the last year of rhGH replacement and in first year after rhGH discontinuation compared with a healthy counterpart. Because some polymorphisms of the GHR could have a role in glucose metabolism and insulin resistance, we also evaluated whether the d3GHR deletion has a role on glucose homoeostasis during and after rhGH withdrawal.

## Methods

This was a single-center longitudinal study conducted at Division of Pediatrics, University of Piemonte Orientale (Novara, Italy). We consecutively recruited 35 GH-deficient (GHD) adolescents at the end of puberty and in the last year of rhGH therapy and 45 healthy age and sex matched adolescents. The recruitment was opened from September 2006 to December 2011 The study was approved by the Ethics Committee of Maggiore della Carità Hospital (Novara) and informed written consent was obtained from all subjects and their parents before study. Patients were eligible if they had an idiopathic isolated GHD at diagnosis, had completed puberty (Tanner stage 4 and 5), the adult height was almost achieved with a bone age similar to the chronologic age and growth velocity near to 2 cm/year, and GHD was not confirmed at retesting (GD, group 1). Patients with coexistent other chronic or endocrine diseases, syndromes, diabetes, tumors or drugs interfering with glucose metabolism were excluded. Healthy control adolescents (CS, group 2) were eligible if they were at the end of puberty, normal-weight, with no history of organic or psychiatric diseases in particular no neurological, endocrine, liver, and kidney abnormalities.

### Study Design and Assays

GHD adolescents were evaluated at fasting for IGF-1, total- (T-c), HDL- (HDL-c) cholesterol, triglycerides (TG), C-peptide and with an OGTT (1.75 g of glucose solution per kg, maximum 75 gr) in the last year of rhGH therapy (T0), and at 6 (T6) and 12 months (T12) after therapy withdrawal. In the year after GH discontinuation the retesting was performed with the GHRH plus arginine test with the adoption of the transition cut-offs [26]. In healthy adolescents the OGTT was performed at baseline.

Impaired fasting glucose (IFG) and impaired glucose tolerance (IGT) were defined according to American Diabetes Association classifications as fasting plasma glucose of ≥100 to 125 mg/dl nmol/l, and as 2-h post-OGTT glucose of ≥140 to 199 mg/dl, respectively. Also the definition of diabetes was performed according to the criteria of the American Diabetes Association [27].

Blood samples during OGTT were drawn for the determination of glucose and insulin every 30 min from 0′ to 120′ min. The area under curve (AUC) for plasma glucose and insulin were calculated by the trapezoidal rule.

Insulin resistance was estimated, in the basal state, by use of the homeostasis model assessment (HOMA-IR) = fasting glucose × fasting insulin/22.5; beta-cell function at fasting was calculated using the formula of HOMA-β = (20 × fasting insulin)/(fasting glucose −3.5). Insulin sensitivity was calculated from the Matsuda [10,000/√(fasting glucose × fasting insulin) × (Gm×Im)] and QUICKI (1/log_10_ fasting insulin+log_10_ fasting glucose) indexes [28].

The area under the curve (AUC) for parameters after OGTT was calculated according to the trapezoidal rule. Delta glucose (ΔG_30–0_) and insulin (ΔI_30–0_) were evaluated as the change in glucose and insulin concentrations from 0 to 30 min. The stimulus for insulin secretion in the increment in plasma glucose as insulinogenic index was calculated as the ratio of the changes in insulin and glucose concentration from 0 to 30 min (INS). Βeta-cell compensatory capacity was evaluated by the disposition index defined as the product of the Matsuda Index and INS (DI) [29]. In addition, each subgroup and all subjects together were divided into four groups according to the 2-h glucose levels,: 1) less than 100 mg/dL, 2) 100–119 mg/dL, 3) 120–139 mg/dL 4) above 140 mg/dL according to the risk to have a lower DI [30].

Plasma glucose levels (mg/dl; 1 mg/dl:0,05551 mMol/liter) were measured by the gluco-oxidase colorimetric method (GLUCOFIX, by Menarini Diagnostici, Florence, Italy). Insulin (µUI/ml; 1 µUI/ml = 7.175 pmol/l) was measured by chemiluminescent enzyme-labelled immunometric assay (Diagnostic Products Corporation, Los Angeles, CA). Sensitivity: 2 µUI/ml. Intra- and inter-assay CV ranges: 2.5–8.3 and 4.4–8.6%.

HbA1c levels were measured by the high-performance liquid chromatography (HPLC), using a Variant machine (Biorad, Hercules, CA); intra- and inter-assay coefficients of variation are respectively lower than 0.6 and 1.6%. Linearity is excellent from 3.2% (11 mmol/mol) to 18.3% (177 mmol/mol).

T-c (mg/dl; 1 mg/dl: 0.0259 mMol/l), HDL-c (mg/dl; 1 mg/dl: 0.0259 mMol/l), TG (mg/dl; 1 mg/dl: 0.0113 mMol/l), and C-peptide (ng/ml) were evaluated using standardized methods in the hospital’s chemistry laboratory. Plasma T-c concentration was measured by esterase and oxidase conversion (Advia 1650, Bayer Diagnostics, Newbury, UK); coefficient of variation (CV) 1.9%. Plasma TG and HDL-c concentrations were measured by enzymatic determination (Advia 1650, Bayer Diagnostics, Newbury, UK); CV 1.7%. LDL-c was calculated by Friedwald mathematical for individuals with TG<150 mg/dl.

Serum IGF-I was measured by Liason automated chemiluminescence analyzer supplied by DiaSorin with a measurement range of 3–1500 ng/ml. Age and gender-reference ranges were used to calculate an IGF1 SDS for each patient.

### Anthropometric Measurements

Height was measured by the Harpenden stadiometer and weight by using electronic scale. Body mass index (BMI) was calculated as body weight divided by squared height (kg/m^2^). Height, weight and BMI were stratified according to Italian growth charts [31]. Height velocity (HV) was calculated from the difference of mean heights obtained from 2 consecutive visits, divided by time between visits, and adjusted to a 12-month interval. Waist circumference was measured with a soft tape, midway between the lowest rib margin and the iliac crest, in the standing position. Hip circumference was measured over the widest part of the gluteal region, and the waist-to-hip ratio was calculated. Systolic (SBP) and diastolic (DBP) blood pressure were measured three times at the left arms by using a standard mercury sphygmomanometer and the mean value was recorded and stratified according to paediatric percentiles of National High Blood Pressure Education Program Working Group on High Blood Pressure in Children and Adolescents [32].

### DNA Extraction and Genetic Analysis

At the end of the clinical protocol, after T12, genomic DNA was isolated from peripheral blood leukocytes by standard methods. The d3GHR polymorphism was detected as described previously [33] based on a multiplex PCR assay with a combination of 3 primers (G1, G2 and G3) that specifically amplify the wild type (935 base pairs bp) and the deleted (532 bp) alleles. Amplification products were visualized by electrophoresis on a 1.5% agarose gel stained with ethidium bromide (Figure S1).

Subjects with at least one copy of the exon-3 deleted GHR (d3/d3 and fl/d3; Del) were grouped together for comparison with full-length homozygote subjects (fl/fl; nDel).

### Statistical Analysis

Data are expressed as mean±SEM. For continuous variables, the variation between groups was compared by means on nonparametric Wilcoxon, Mann-Whitney U, or chi-square tests, where appropriate. Trends were assessed by nonparametric Friedman test. A correlation analysis was performed using the Pearson’s correlation test with a logarithmic transformation when necessary.

Statistical significance was assumed for p<0.05. All statistical analyses were performed with SPSS for Windows version 17.0 (SPSS INC; Chicago, IL, USA).

## Results

### GHD before and after GH Discontinuation

Of the 35 GHD enrolled subjects, 23 performed two OGTTs and completed the study. Of the 45 CS, 5 subjects had discomfort during OGTT and were excluded.

Clinical characteristics of CS and GHD at T0 and T12 are reported in Table 1 and 2. GHD subjects had received GH replacement therapy for 7.3±2.0 years. The last dose of GH was 12.0±0.5 mg/week. The GH peak at the Arginine+GHRH test was of 72.0±5.6 ng/ml.

**Table 1:** Clinical parameters of growth hormone deficient (GHD, group 1) children at the end (T0) of rhGH therapy (T0), after 12 months rhGH withdrawal (T12) and of control subjects (CS, group 2).

	GHD (group 1)T0	GHD (group 1)T12	CS (group 2)
**N°**	23	23	40
**M/F**	12/11	12/11	22/18
**IFG/IGT**	1/2	0/0	0/0
**Age (yr)**	15.9±0.2	17.9±0.2	15.4±0.2
**Weight (kg)**	55.4±1.8	58.6±2.1	61.3±1.4
**Centile weight**	23±2.1	25±2.1	27±1.5
**Height (cm)**	163.5±1.7^a^	166.1±1.2^c^	173.7±1.2^a,c^
**SDS height**	−0.9±0.1^a^	−1±0.2^c^	1.1±0,1^a,c^
**BMI (kg/m^2^)**	20.9±0.5	21.2±0.5	20.4±0.2
**HV (cm/yr)**	1.5±0.4	1.0±0.1	–
**Waist (cm)**	72.8±1.2^a^	77.4±1.4	79±0.8^a^
**SBP (mmHg)**	119.7±2.1^b^	119.4±3	124.4±1.3^b^
**DBP (mmHg)**	77.2±1.4	78.0±2.6	79.8±1.2

Abbreviations. BMI, body mass index; HV, height velocity; IFG, impaired fasting glucose; IGT, impaired glucose tolerance; SBP: systolic blood pressure; DBP: diastolic blood pressure, SDS: standard deviation score.

Data are expressed as mean±SEM. a: p<0.0001 GHD T0 vs CS; b: p<0.05 GHD T0 vs CS; c: p<0.0001 GHD T12 vs CS.

**Table 2:** Glucometabolic parameters of growth hormone deficient (GHD, group 1) children at the end of rhGH therapy (T0) and 6 (T6) and 12 (T12) months after rhGH withdrawal.

	T0	T6	T12	p^for trend^
**Fasting glucose (mg/dl)**	83.6±1.7	84.5±1.8	84.1±1.8	NS
**30-min plasma glucose (mg/dl)**	145.5±5.7	138.3±5.4	127.2±5.9	<0.05
**2-h plasma glucose (mg/dl)**	110.1±4.2	98.6±3	92.5±5	<0.01
**AUC Glucose 0-120 min**	11592.4±521	11273.4±381	10691.8±613	NS
**Mean Glucose (mg/dl)**	114.7±4.2	111.1±3	105.4±4.9	NS
**ΔG_30-0_**	59.9±4.8	53.2±4.9	42.9±5.5	<0.05
**Fasting insulin (µUI/ml)**	10.5±1.1	8±0.7	7.7±0.6	<0.05
**30-min plasma insulin (µUI/ml)**	99.4±12	80.6±11.6	59.8±9	<0.01
**2-h plasma insulin (µUI/ml)**	59.6±6.8	40.9±5.4	34.2±4.8	<0.01
**AUC Insulin 0–120 min**	7875.6±915	6554.4±864	4882.1±591	<0.01
**Mean Insulin (µUI/ml)**	66±7.2	53.3±6.4	40.6±4.3	<0.01
**ΔI_30-0_**	88.8±11.2	72.5±11	52.2±8.6	<0.01
**HOMA-IR**	2.2±0.2	1.7±0.1	1.6±0.1	<0.05
**HOMA%β**	205.2±23.8	148.5±15.9	148.3±16.2	<0.05
**INS**	1.6±0.2	1.3±0.1	1.2±0.2	<0.05
**DI**	6.8±0.7	7.2±0.6	7.9±1.0	NS
**C-peptide (ng/ml)**	0.21±0.05	0.19±0.04	0.36±0.05	P<0.01
**T-c (mg/dl)**	141.6±4.6	136.2±4.5	136.8±6.9	NS
**HDL-c (mg/dl)**	52.2±2.0	46.2±1.7	48.2±2.6	NS
**LDL-c (mg/dl)**	76.1±3.6	77.5±3.2	76.6±5.7	NS
**TG (mg/dl)**	60.7±4.1	62.7±6.2	59.2±5.3	NS
**IGF-1 (ng/ml)**	629.6±38	356.8±17	332.6±25	<0.0001
**IGF-1 SDS**	1.21±0.19	-0.01±0.06	-0.15±0.17	<0.0001

Abbreviations. DI, disposition index; INS, insulinogenic index; AUC, area under the curve; ΔG0-30: delta glucose;; ΔI0-30: delta insulin; HDL-c, HDL-cholesterol; LDL-c, LDL-cholesterol; T-c, total-cholesterol; TG, triglycerides. Data are expressed as mean±SEM. The significance among the three measures (T0, T6 and T12) was calculated by Friedman test.

One GHD subject showed IFG at T0 and T6 and two subjects IGT at T0. No glucose alterations were found at T12. The distribution of post-OGTT 2hrs glucose levels at each time point was reported in Table 2. HbA1c levels were 5.3±0.1% (34.0±1.0 mmol/mol) in the last year of therapy. No differences in waist, waist/hip ratio, BMI, SBP, DBP, LDL-c and TG were shown in GHD among visits. T-c and HDL-c levels were lower at T6 than T0, and HDL-c levels also at T12 than T0 in GHD although the trend among visits was not significant (Table 2).

Basal glucose levels were similar among the 3 times. Fasting insulin (p<0.05), HOMA-IR (p<0.05) insulin and glucose levels at each time point after OGTT (p<0.01), ΔG_0–30_ (p<0.05) and ΔI_0–30_, insulin mean and insulin AUC (p<0.01) progressively decreased from T0 to T12. Conversely, C-peptide (p<0.0001), Matsuda and QUICKI indexes increased from T0 to T12 (p<0.05). Despite higher insulin resistance during the last year of GH treatment, a compensatory insulin secretion was recorded: HOMA-β and INS were higher at T0, and progressively decreased at T6 and T12 (p<0.05), with a stable DI. Also IGF1 levels and IGF-I SDS decreased from T0 to T12 (p<0.0001) (Table 2).

A trend to decrease in DI across 2 h glucose groups (<100; 100–119; 120–139; >140 mg/dl) was observed and maintained at each time point in GHD subjects; T0 (8.7±1.4; 7.2±1.1; 5.2±1.4; 3.2±0.8, respectively; p<0.01; χ^2^ 12.156), T6 (7.5±1; 7.0±0.9; 5.6±0.1, respectively; p<0.05; χ^2^ 6.695) and T12 (8.7±1.4; 7.6±1.7; 3.6±1.3, respectively; p<0.05; χ^2^ 6.593).

HOMA-IR (p<0.03), HOMA-β (p<0.002), QUICKI (p<0.03), Matsuda indexes (p<0.002), fasting (p<0.05) and at 120 min glucose (p<0.01) were worse in GHD subjects at T0 than in CS. No differences in metabolic parameters were shown between CS and GHD at T12 (Table 1).

### GHD before and after GH Discontinuation According to GHR Genotype

By analysing the GHD group as a whole, we observed a huge dispersion in INS and DI as mean±SD. Indeed, we addressed to understand whether d3GHR has a role, according to some literature suggestions (9,10). All the GHD subjects were analysed for the GHR genotype, meanwhile 5 out of 40 CS withdrawn the consent to perform this analysis.

The frequencies of the tree genotypes, d3/d3, fl/d3 and fl/fl were respectively 9% [2], 35% [8], 56% [13] in GHD. Allele frequencies were not different from those observed in the 35 CS (Figure S1). Because frequency, the participants were therefore divided into two groups, those homozygote for the fl/fl alleles (Del) and those hetero- and homozygote for the deleted isoform combined (nDel).

Del and nDel GHD had similar anthropometric and metabolic parameters at T0 with exception of HDL-c which was higher in Del GHD (56.5±1.9 vs 49.1±3.0 mg/dl, p<0.01).

At T6, Del GHD showed lower glucose levels at baseline and after OGTT than nDel GHD (fasting glucose: 78.7±1.8 vs 87.5±2.9 mg/dl, p<0.01; T120 glucose: 90.7±4.6 vs 101.0±3.5 mg/dl, p<0.01; AUC 9703.0±476.6 vs 12316.7±447.7 mg/dl*h, p<0.01 and mean glucose 97.1±3.6 vs 118.5±2.8 mg/dl, p<0.0001). DI was higher in Del than nDel GHD (10.4±1.0 vs 5.4±0.4; p<0.01) without differences in INS, HOMA-IR and HOMA-β.

**Figure 1:**
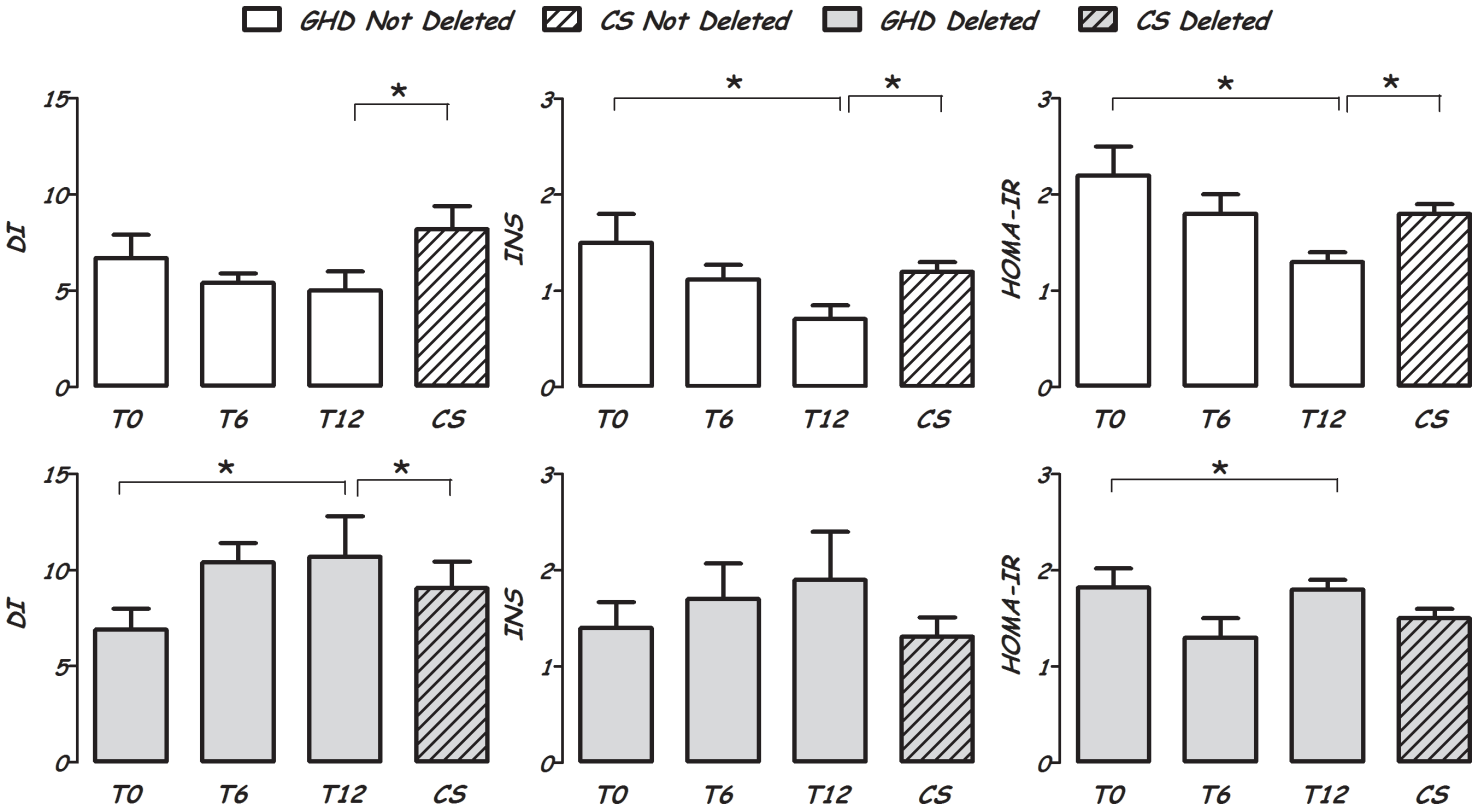
HOMA-IR, Insulinogenic (INS) and disposition (DI) index in GH deficient (GHD, group 1) and healthy (CS, group 2) adolescents with (Del, 27 subjects) and without (nDel, 31 subjects) the GH receptor (GHR) exon 3 deletion (d3GHR). GHD adolescents are evaluated in the last year of therapy (T0) and after six (T6) and twelve (T12) rhGH withdrawal. Data are expressed as mean±SEM. The significance among the three measures (T0, T6 and T12) was calculated by Friedman test. The significance between GHD and CS was calculated by Mann-Whitney U test. *p<0.05.

At T12, Del GHD showed lower glucose AUC (9449.0±552.4 vs 11771.0±1068.6 mg/dl*h; p<0.05) and ΔG_30–0_ (29.5±4.5 vs 53.0±9.0 mg/dl; p<0.05), and higher fasting insulin (9.0±0.8 vs 6.4±0.8 µUI/ml; p<0.05),HOMA-IR (1.8±0.1 vs 1.3±0.2; p<0.05) and C-peptide (0.39±0.09 vs 0.24±0.03 ng/ml; p<0.04) than nDel GHD. Moreover, Del GHD had INS (1.8±0.5 vs 0.7±0.1; p<0.01) and DI (10.7±1.8 vs 5.0±1.0; p<0.01) higher than nDel GHD. nDel GHD showed a progressively decrease from T0 to T12 in HOMA-IR, HOMA-β, INS (p<0.05) and DI (not significant) with stable C-peptide levels. Conversely, Del GHD showed a gradual increase in DI (p<0.05), INS (not significant), and C-peptide (0.20±0.08 vs 0.39±0.09; p<0.01) with a HOMA-IR decreasing from T0 to T6 with a slight increase to T12 (p<0.01 for trend) (Figure 1).

Del GHD presented stable higher HDL-c levels than nDel GHD at any time point, whereas T-c, LDL-c and TG remained similar. T-c levels decreased from T0 to T12 in nDel GHD (141.0±7.4 at T0; 137.2±7.5 mg/dl at T6, 133.5±8.4 mg/dl at T12; p<0.0001).

At T12, Del GHD presented lower fasting glucose (83.5±2.2 vs 88.0±2.2 mg/dl p<0.03), and higher fasting insulin (9.0±0.8 vs 6.8±0.6 µUI/ml; p<0.03), HOMA-β (167.7±20.1 vs 96.9±10.1; p<0.003), and DI (10.7±1.8 vs 9.1±1.0 p<0.05) than Del CS. Conversely, at T12 nDel GHD presented lower HOMA-IR (1.3±0.2 vs 1.8±0.1 p<0.03), INS (0.7±0.1 vs 1.2±0.1 p<0.04), and DI (5.0±1.0 vs 8.1±1.2 p<0.04) than nDel CS. Del and nDel CS had higher C-peptide levels with respect to their GHD counterpart (p<0.001).

### Correlations

In GHD subjects during GH therapy, both INS and DI were correlated with GH weekly dose (r: −0.399; p<0.05 and r: −0.529; p<0.01, respectively). Correlation was maintained when correcting for IGF-1 (INS r: −0.392, p<0.05; DI r: −0.526, p<0.01p<0.01) or IGF-SDS (INS r: −0.398, p<0.05; DI r: −0.531, p<0.01) which also weights for age and gender. No correlations were recorded after GH discontinuation.

IGF-1 levels and IGF-SDS were correlated with HOMA-IR at T6 (r: 0.518 and r: 0.514, p<0.01) and T12 (r: 0.518 and r: 0.518, p<0.01), and also with QUICKY and Matsuda indexes at T6 (QUICKI, r: −0.495 and r: −0.476, p<0.03; Matsuda index, −0.443 and r: 0.302; p<0.05) and T12 (QUICKI, r: −0.611 and r: 0.–609, p<0.02; Matsuda index, −0.543 and −0.359; p<0.05).

## Discussion

A large number of studies have addressed the metabolic consequences of adult GHD, in contrast studies investigating glucose and insulin metabolism at the end of GH therapy in not confirmed GHD adolescents during the transition phase are lacking. The lack of published data reflects the clinical practice of stopping the follow up in adolescents at the achievement of adult height and with not confirmed GHD at reevaluation. This practice makes a gap in the understanding of GH biology and of long-term safety of rhGH treatment. In the present study, we observed that after 1 year of rhGH withdrawal metabolic parameters are similar between not confirmed GHD and matched control subjects. Moreover, during GH treatment, higher insulin-resistance at fasting and lower insulin-sensitivity during OGTT are associated with higher HOMA-β and insulinogenic index and a stable DI in the group as a whole. The d3GHR deletion may have a role on the metabolic risk with a more pronounced reduction of HOMA-IR and compensatory insulin secretion in nDel GHD, and with lower glucose levels and a higher increase in DI and fasting C-peptide in Del GHD at the end of the treatment.

First of all, we showed that whether glucose impairment and/or insulin resistance occurred during the treatment with rhGH, these are transient and quickly restored also in not confirmed GHD adolescents, as already demonstrated in literature in adolescents with persistent GHD [19], [34]. Although puberty is ongoing, insulin resistance at fasting and insulin sensitivity during OGTT progressively return to similar levels to age, puberty, and weight matched healthy adolescents. On the other hand, it is interesting that GHD treated adolescents have the well-known higher insulin resistance at fasting [35], [36] but it is coupled with a compensatory insulin secretion both at fasting, measured as HOMA-β, and during OGTT, measured as INS with also a stable DI. Indexes of insulin sensitivity (Matsuda index) and early insulin responses to oral glucose (INS) that were derived from baseline and follow-up OGTTs did not appear to be significant predictors for the development of type 2 diabetes. In contrast, the baseline DI, which significantly assesses beta-cell function in the context of insulin sensitivity, predicts the risk of deteriorating glucose tolerance in youths [30]. Thus, insulin secretion can be truly evaluated only in relation to the degree of insulin sensitivity. Indeed, our data on DI suggest that whether the treatment with GH has a detrimental role on insulin resistance because it stimulates lipolysis, in youths pancreas maintains a beta-cell compensatory capacity and is able to answer to a relative hyperglycemia with a higher insulin secretion which counterbalances the GH inhibited insulin-stimulated glucose uptake in the muscles without impact on future type 2 diabetes risk in those healthy [4], [5]. Similarly, although M values at euglycemic hyperinsulinemic clamp were higher after a short rhGH withdrawal, in GHD treated young adults M values were similar than matched controls [36]. Also in small for gestational age GH treated adolescents, insulin sensitivity decreased but DI remained stable at puberty [37]. We could hypothesize that the preserved beta-cell function at early age protects from type 2 diabetes and that when DI progressively decreases with age and obesity a sustained glucose impairment arises more likely as in adults. A direct role of GH on insulin antagonism is supported by the negative correlation between GH weekly dose and INS and DI, suggesting that the pancreas compensation we observed is secondary to the peripheral detrimental action of GH.

Studies conducted in adults and children suggest that insulin resistance and glucose alteration are more frequent in GH treated subjects with risk factors like age, obesity, an adverse metabolic profile before therapy, syndromes or history of tumors [16]. Genomic profiles, like GHR polymorphisms could be inserted in this list. The d3GHR deletion is the most investigated polymorphism of GHR in both healthy subjects and patients with GHD or other diseases such as acromegaly or type 2 diabetes [38]. The d3GHR variant consisting of genomic exon 3 deletion has been linked with increased receptor activity due to an enhanced signal transduction. Its biological role on glucose metabolism is still controversial. Data derived by the Stockholm Diabetes Prevention Program suggest the homozygosity for the d3GHR allele as preventive of type 2 diabetes in adults. However, when other factors cause overt type 2 diabetes, the d3GHR allele confers a phenotype indicative of risk for metabolic disorders [10]. In Chinese obese children fasting insulin, HOMA-IR, and lipid profile were significantly lower in the homozygous and heterozygous d3GHR group than in the full-length GHR group [39]. Moreover, the presence of at least one d3GHR allele was associated with higher insulin secretion for a given degree of insulin sensitivity and with a higher disposition index in healthy normal weight children and adolescents during puberty in the COPENHAGEN puberty study [11].

All these data are in agreement with our observation that glucose levels are lower and DI, fasting C-peptide and nearly to significance INS, were higher in Del GHD adolescents after stopping therapy, suggesting that GHR exon 3 deletion gene polymorphism may play a role in modulating insulin secretion to a glucose challenge and peripheral insulin activity at least during periods of high endogenous GH secretion like puberty. Higher fasting C-peptide in subjects with at least one copy of the exon-3 deleted GHR than those with the full length one were also demonstrated in adults [10]. However, the changes are apparent after rhGH withdrawal in our population. This result is in line with that of Giavoli *et al*. who recorded a higher prevalence of impaired glucose tolerance after both 1 and 5 years of rhGH therapy in GHD adults positive for the d3GHR [9]. The enhanced activity of the d3GHR isoform may mediate the metabolic effects of rhGH on glucose homeostasis. We can speculate that a more pronounced GH activity has a strong lipolytic effect and insulin antagonism. As a consequence, a worse glucose homeostasis in adults and, more likely, a partial deterioration in peripheral insulin activity, reflected by DI, in adolescents become apparent. This hypothesis is fully in agreement with the fact that in healthy people when other factors cause overt type 2 diabetes, the d3GHR allele is not protective but confers a more impaired metabolic phenotype [10]. Because normal adolescents with d3GHR have higher DI during puberty and GHD unconfirmed subjects present an increase in DI after rhGH withdrawal, a threshold for the metabolic GH activity is suggested, in agreement with studies which failed to record alterations in glucose metabolism for treatments with rhGH very low doses [40].

GH plays an important role in the regulation of lipoprotein metabolism. In patients with d3GHR we also observed higher HDL-cholesterol levels during rhGH treatment and after rhGH withdrawal. These findings are in line with data in healthy and hypertensive adults [41] and support the idea that sequence variations in the GHR may have important effects on metabolic phenotype as a whole. Accordingly, other polymorphisms in the GHR have been shown to modify HDL cholesterol concentration also in hyperlipidaemic patients [42].

It has to note that our subjects progressively decreased IGF-I levels after rhGH withdrawal but remained in the middle part of the normal range. Higher IGF-I levels in the last year of rhGH treatment were due to the treatment and, in absence of a confirmed GHD during the transition phase, their levels quickly restored in the middle of normality. Interestingly, insulin resistance and sensitivity negatively correlated with them only after the withdrawal of the therapy. This is adequate with previous study that attest the U shape relation of IGF-I with the risk of diabetes is evident in a healthy population when confounders are removed [43].

There are limitations in the present study. First is that we did not perform an intravenous glucose tolerance test (IVGTT). The IVGTT is validated and more reproducible than the OGTT, because independent by confounding factors as the incretin effect is. However, the IVGTT only describes an experimental model and is far to be used in clinical practice. We decided to use the OGTT instead of IVGTT because OGTT describes the post-meal dynamics which is of interest for the GH physiology being GH a hormone that principally acts in the post-absorptive phase [1]. Moreover, we chose the OGTT-derived indices of insulin sensitivity and secretion because they correlate well with clamp measures in children [44], [45], are easily calculated in epidemiological or intervention studies, and DI calculated by the OGTT has been demonstrated to predict diabetes development in adults [24]. Furthermore, the OGTT is the test that is classically performed in the clinical practice during rhGH treatment being simpler and cheaper than IVGTT. Moreover, we did not evaluate C-peptide during the test but only at fasting. The second limitation is that we were unable to demonstrate a gene dosage effect with respect to the d3GHR allele because the group of homozygous subjects was too small to analyse them separately. A study in a wider cohort could address this question. However, many published studies on the d3GHR also in wider populations [9]–[11] grouped together subjects bearing at least one copy of the exon-3 deleted GHR according to the hypothesis of the dominant model [46].

On the whole, in not confirmed GHD adolescents the fasting deterioration in glucose homeostasis is efficaciously coupled with a compensatory insulin secretion and activity for the degree of insulin-sensitivity at glucose challenge also in puberty. In adolescents during puberty, the presence of at least one d3GHR allele, despite a slightly higher fasting insulin resistance, is associated with lower glucose levels and higher HOMA-β, fasting C-peptide, HDL-cholesterol and DI at OGTT after rhGH withdrawal. Because a more pronounced activity of the d3GHR isoform, a major risk to develop an impairment in glucose homeostasis during the treatment could be hypothesized and future studies should address this question. OGTT should be routinely performed in clinical practice to better stratified GHD subjects during the treatment. Screening for the d3GHR in GHD treated subjects, also in childhood may help physicians to follow and phenotype patients during the treatment.

## Supporting Information

Figure S1 **Analysis of the GHR exon 3 polymorphism.** a) Schematic representation of the multiplex PCR assay used to detect the GHR exon 3 polymorphism. One forward (G1) and two reverse primers (G2 and G3) were used. Primers G1 and G3 (this located within exon 3) are designed to detect the GHR-fl allele by the amplification of a 935 bp fragment (the 3248 bp fragment is non amplified under the conditions used); primers G1 and G2 allow the amplification of the GHR-d3 by producing a 532 bp fragment. b) Genotyping of the GHR exon 3 polymorphism. The presence of a 935 bp band indicates the genotypes homozygous for GHR-fl (#1,2,3,6,8,10,11,12) the presence of a band of 532 bp indicates the genotype homozygous for GHR-d3 (#4) and the presence of both the bands indicates the herozygotes (#5,7,9,13). A 100 bp ladder is used as a molecular weight marker. c) Distribution of the genotype in GHD and control (CS) subjects. Five out of 40 CS did not give the consent to perform the genetic analysis.
(TIF)
