# Supplementary figures and images for: Effects of Growth Hormone (GH) Therapy Withdrawal on Glucose Metabolism in Not Confirmed GH Deficient Adolescents at Final Height
Source: PLoS One. 2014 Jan 30;9(1):e87157. doi: 10.1371/journal.pone.0087157 (PMC3907518; doi:10.1371/journal.pone.0087157)

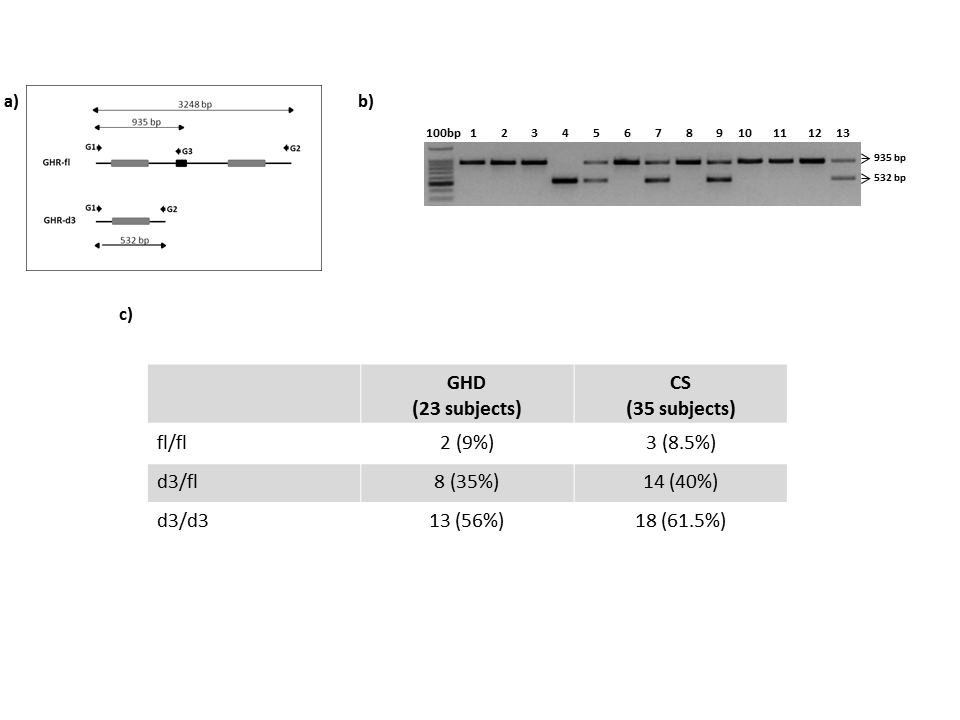

Supplement: Figure S1 — Analysis of the GHR exon 3 polymorphism. a) Schematic representation of the multiplex PCR assay used to detect the GHR exon 3 polymorphism. One forward (G1) and two reverse primers (G2 and G3) were used. Primers G1 and G3 (this located within exon 3) are designed to detect the GHR-fl allele by the amplification of a 935 bp fragment (the 3248 bp fragment is non amplified under the conditions used); primers G1 and G2 allow the amplification of the GHR-d3 by producing a 532 bp fragment. b) Genotyping of the GHR exon 3 polymorphism. The presence of a 935 bp band indicates the genotypes homozygous for GHR-fl (#1,2,3,6,8,10,11,12) the presence of a band of 532 bp indicates the genotype homozygous for GHR-d3 (#4) and the presence of both the bands indicates the herozygotes (#5,7,9,13). A 100 bp ladder is used as a molecular weight marker. c) Distribution of the genotype in GHD and control (CS) subjects. Five out of 40 CS did not give the consent to perform the genetic analysis. (TIF) [file pone.0087157.s001.tif]
